# Dr. John Shaw Billings

**Published:** 1913-04

**Authors:** 


Dr. John Shaw Billings died of pneumonia in New York March 11, aged 73. He was graduated in medicine from the Medical College of Ohio, Cincinnati, in 1860, entered the U. S. Army as Assistant Surgeon in 1862, and gained the rank of Lt.-Col. in 1894, being retired in 1895. His great work was the building up of the Army Medical Museum and the Index Catalogue. His accomplishments were recognized by honorary degrees in medicine from Munich and Dublin, D.C.L. by Oxford, and LL.D. by several universities.

